# Community Care Centre (CCC) as adjunct in the management of Ebola Virus Disease (EVD) cases during outbreaks: experience from Sierra Leone

**DOI:** 10.11694/pamj.supp.2015.22.1.6521

**Published:** 2015-10-10

**Authors:** Olushayo Olu, Martin Cormican, Kande-Bure Kamara, Waqar Butt

**Affiliations:** 1 World Health Organization (WHO) Inter country Support Team for Eastern and Southern Africa, Zimbabwe; 2 School of Medicine, National University of Ireland Galway, Galway, Ireland; 3 World Health Organization (WHO) Geneva, Switzerland

**Keywords:** Ebola Virus Disease, Community Care Centre (CCC), Sierra Leone

## Abstract

Community Care Centres (CCCs) represent an innovative response to the containment of infection and the care of those infected in the context of an an Ebola Virus Disease (EVD) outbreak of unprecedented scale. This paper describes the implementation of this response in the Port Loko district of Sierra Leone in the last quarter of 2014. CCCs were effective in encouraging EVD patients to come forward, thus removing risk of transmission to their families and communities however there is significant scope for improvement in care for patients in the centres if the model is applied in future outbreaks of infectious disease. Changes in lay out of the centres, in staff training and support, in logistics and patient education are recommended.

## Introduction

Sierra Leone has borne a significant burden of the ongoing EVD outbreak in West Africa. As of 25th February, 2015 the country had reported a cumulative total of 11,301 cases and 3,461 deaths (CFR 31%) making it the worst affected country in the history of the disease [1]. Due to the scale of this outbreak, implementation of key EVD prevention and control strategies such as timely identification and isolation of all cases, follow up of all contacts, safe and dignified burial of the dead and community mobilization have proven difficult. At the height of the outbreak in October 2014, the daily EVD caseload by far outstripped the available bed capacity especially in the two worst affected districts of Western Area and Port Loko. In the absence of admission space in the EVD holding and treatment centres, several cases which had been triaged and classified as either suspected or probable remained in their homes thus increasing the risk of secondary infection of their families, close associates and also increasing community transmission of the disease which has been a hallmark of this outbreak [2]. Hitherto, the management of EVD cases is usually conducted in EVD Treatment Centres (ETC) where high levels of infection prevention and control are practised and there is adequate staffing and relatively large bed capacities [3]. However, the vast number of cases reported in this outbreak has seen these conventional treatment centres fill up quickly, leaving the health stakeholders with very few options for effective EVD case isolation. For a period of time EVD patients were managed in designated areas within general public hospitals such as the Port Loko General Hospital or community health centres which were wholly or partly allocated to this function and called holding centres. The use of the public hospitals and community health centres for this purpose contributed to the collapse of general health services in the country. Much of the available space and resources in these facilities were consumed in the care of Ebola patients and the fear of Ebola among community members meant that they were often unwilling to access the little general health services that were still being provided. Faced with these unprecedented pressures, in September 2014, WHO in consultation with the Ministry of Health and Sanitation (MOHS) of Sierra Leone and other partners developed a novel strategy known as Community Care Centres (CCCs). The goal was to rapidly increase the available capacity for EVD case isolation and management at the community level. Simultaneous roll out of the strategy was implemented in Liberia [4]. The first four CCCs in Sierra Leone were sited in Kabantama, Ferodugu, Lunsar, and Kamasundu villages of Port Loko district and became operational in early November 2014. This paper presents a critical perspective on the CCC experience in Port Loko district with some recommendations as to how the strategy could be improved and adapted for use in future outbreaks of EVD.

### The CCC Strategy

A CCC is a community-based Ebola isolation unit where suspected and probable cases of EVD are isolated and provided with basic health care as they await their confirmatory test results. They also serve as places where confirmed cases can commence early treatment as they await transfer to bigger ETCs. They are small, low technology, mainly staffed by nurses and community health workers and can accommodate 8 to 10 (maximum 15) patients. They focus on supportive management of patients; invasive procedures (except for collection of blood samples for EVD confirmatory test) are discouraged to reduce the risk of health facility acquired infection. The objectives of CCCs are to among others complement conventional ETCs by early isolation of EVD cases as close as possible to their communities and to improve access to Ebola care among the general populations especially for those in rural areas. The basic package of care offered at CCCs include presumptive treatment for malaria, supportive therapy such as management of fevers, body pains etc., provision of Oral Rehydration Solution (ORS), food, water and psychosocial care among others [5]. The CCC strategy also incorporates components of community engagement, participation, safe and dignified burials.

### Organization of the CCC Infrastructure

CCCs were of two forms; frequently they were improvised around existing permanent structures, for instance the CCC in Lunsar was established within a hospital. In other circumstances CCCs consisted entirely of temporary shelters (tents) constructed on a new site. The centres were clearly identifiable at a distance as CCCs, were peripheral to or outside of a village, they required access to a water source for cleaning and washing, and were well demarcated from the surrounding community by a perimeter fence usually made of plastic sheeting. The CCCs had separate access points for staff arriving for work and for patients presenting for assessment. The centres had triage areas constructed with at least one metre separation between patient and staff areas. The triage area opened into a patient area (red zone). Movement to and from the triage zone to the red zone was variably controlled and frequently allowed movement in both directions. The red zone consisted of two or more separate spaces, either rooms within existing permanent structures or tents which were intended as cohort areas for two different categories of patients, those most infectious and those likely to be less infectious. There was generally no effective barrier to movement between these cohort areas within the red zone. The red-zone included patient latrines and shower facilities. In some cases communal traditional thatched shelters were provided in each cohort area for patient comfort. The red-zone included soak pits for effluent from showers. There were three exits from the red-zone, one to a mortuary area, one for those discharged from the CCC to the community and one through a PPE doffing area for staff leaving the red zone. The staff entrance to the CCC opened into the “green zone” where there were staff facilities including latrines and changing area. These facilities were in existing buildings or temporary tents. The green zone also included storage areas and staff rest areas. There is an entrance, typically gated, from the green zone into the red zone. Typically close to the exit from the red zone there was a pit for disposal of used PPE and other waste.

### Operational Practice in the CCC

Patients presented to a triage area either on foot or by ambulance. Staffs were expected to assess patients presenting by application of a triage algorithm. Those assessed as requiring admission then entered the patient area (red zone). Patients were confined to the red zone until such time that a blood test for detection of Ebola virus was available. The interval from admission to receipt of a laboratory report varied from approximately three to seven days. Patients who were well enough could move freely within their cohort area, sometimes between different cohort areas within the red zone and sometimes between the red zone and the triage area. Typically staff entered the red zone 3 times per day (morning, afternoon and evening) for periods of 30 to 60 minutes on each occasion. During the visits the condition of patients was observed, ORS, water and food was provided and cleaning performed. When patients’ test reports were received those who tested positive were transferred to EVD treatment centres as soon as possible and those with reports of EVD not detected were given a certificate and allowed to leave or provided with transport home.

### Key Observations and Lessons Learnt from Implementation of the CCC Strategy

The CCC model was implemented rapidly in response to unprecedented pressures with a view to controlling ongoing community spread of EVD. Despite concerns raised by several partners about conducting EVD case management in such low technology setting, recent scientific evidence and field experience showed that the strategy has considerable potential for EVD outbreak prevention and control. Kucharski et al. in their recently published article demonstrated that CCCs could reduce EVD transmission in the community if well managed and all things are equal [6]. Logan et al also suggested decentralization of EVD case management as a strategy to scale up EVD patient care based on experience of using CCCs in Liberia [3]. Our field experiences and observations in Port Loko district did show that the strategy had a number of strengths. Firstly, we observed that introduction of the strategy facilitated better community engagement, participation and ownership of the EVD outbreak response effort thus contributing to timely identification and isolation of EVD cases in the areas where they were rolled out. Secondly, the community participation in the process provided a good opportunity for community education and mobilization for action. Thirdly, the siting of the CCCs in the local communities facilitated easy access for sick patients and also allayed the often expressed fears of families that they do not know exactly where their sick relatives were being treated. Fourthly the CCCs eased pressure on the district general hospital, much of which had been re-designated for isolation of suspect Ebola patients (with major impact on maternity services) and on the only large dedicated Ebola management facility in the district at that time (based at Maforki, outside of Port Loko town).

The concept was however not without its challenges. The small size of the units meant that they could only admit limited number of patients whilst the use of cadres of health workers with lower levels of qualification such as community health workers meant that more intensive training, support supervision and monitoring of the infection prevention and control practices was required. Although training was provided on site before opening of CCCs, ongoing training and close supervision of their operation was limited by the relatively remote locations and by the limited number of available IPC specialists. There were also significant supply chain issues resulting in intermittent shortages of drinking water, oral rehydration solution and items of personal protective equipment. Observation of practice within the CCCs suggests that messages to health care workers regarding self-protection at work were well received but balancing this requirement with the need to provide an appropriate level of patient care was less satisfactory. Our observations indicated that even in full PPE many staff would still avoid even minimal physical contact with patients who were too weak to stand or walk and too weak to drink ORS. The force of the message regarding self-protection may also have contributed to the long periods during which no health care workers were present in the red zone leaving debilitated patients to care for themselves or dependent on informal care from other patients. In practice our observations showed that half or more than half of patients admitted to CCCs may not have had EVD at the time of admission. Thus an unintended consequence of the CCC model was that many patients who did not have EVD were potentially exposed to the risk of acquiring EVD in the CCCs for extended periods of up to seven days or more, however this was also the same in the conventional holding centres. The provision of staff supervision in patient areas (which in many cases included minors) and of delivery of an appropriate level of care for critically ill patients with the CCC model was problematic because of the limited time during which staff were present in the red zone and the over whelming emphasis on self-protection in training. Our observations are based on implementation of the CCCs in rural and semi-urban settings; its applicability to urban settings where the community structures are different and transmission (and patient load) are likely to be higher may be tricky due to two main reasons. Firstly since the strategy is based on the principles of community ownership and participation and given that such structures are not well defined in urban centres, community participation in the process may be limited. Secondly, the limited number of beds in the CCC may not be able to cope with the patient load in a high transmission outbreak in an urban area.

## Conclusion

In conclusion, there is some evidence that the use of CCCs to isolate EVD patients in defined areas may have contributed to control of transmission in Port Loko, however there were unintended consequences associated with its roll out. Furthermore, a recent rapid assessment of the CCC concept which was conducted in six districts of Sierra Leone also highlighted key benefits of the concept [7]. The use of cohort as distinct from individual patient isolation method in relation to a disease which is communicable, has a very high mortality and for which there is no specific treatment or prophylaxis available is a major concern. If it is accepted that the intervention was of value in removing infected patients from the community there is an ethical concern about the welfare of non-infected patients admitted to the CCCs [8]. We believe that CCC strategy has potentials in the prevention and control of future EVD outbreaks and could be a useful addition to EVD outbreak management package. However, a number of actions would be required to improve upon the strategy. Further definition of criteria for its use, guidelines for its roll out, management, supervision, monitoring and evaluation are required. In this regard, it would be appropriate to explore the practicality of using low cost, easily erected compounds built around individual patient tents and toilet facilities to isolate EVD patients in CCCs. The training and supports for staff recruited from the community should be improved. Training needs to communicate better balance between the priority of staff protection and the need for patient care. An effective system of insurance for staff to provide some financial security for their families in the event of their acquiring EVD in the course of their work may also enable staff to manage their fears more effectively. A policy to ensure that patients are educated on the risks associated with their admission into CCCs and how they can ensure minimal interpersonal contact during their admission should be included in the CCC guidelines. Continuity of supply of critical items must be assured as interruption of access to drinking water and ORS, in particular may have profound consequences for survival of affected patients. The allocation of limited CCC capacity to patients assessed as critically ill based on clinical status, rather than laboratory test results, could be expected to contribute to improved outcomes and reduced period of time during which non-infected patients are cohorted with highly infectious “wet patients”. Mechanism for following up discharged uninfected patients to determine if some had acquired infection in the CCC would be useful. Finally, further epidemiological studies to provide scientific evidence of the impact of the CCC strategy on EVD transmission would useful.
